# Comparison of clinical characteristics and vestibular function test results in patients with vestibular migraine and Menière’s disease

**DOI:** 10.1016/j.bjorl.2023.05.001

**Published:** 2023-05-16

**Authors:** Yuexia Wu, Xia Ling, Ning Song, Shuangmei Yan, Wenting Wang, Xu Yang, Ping Gu

**Affiliations:** a The First Hospital of Hebei Medical University, Department of Neurology, Shijiazhuang, China; b Peking University Aerospace School of Clinical Medicine, Aerospace Center Hospital, Department of Neurology, Beijing, China; c Peking University First Hospital, Department of Neurology, Beijing, China; d The First Hospital of Hebei Medical University, Department of Vertigo Center, ShiJiazhuang, China

**Keywords:** Vestibular migraine, Menière's disease, Vestibular symptoms, Vestibular tests, Motion sickness

## Abstract

**Objectives:**

The differentiation between Vestibular Migraine (VM) and Meniere's Disease (MD) is difficult because of overlapping symptoms. The study aimed to compare the clinical characteristics and vestibular function test results between VM and MD patients.

**Methods:**

Seventy-one patients with definite VM and 31 patients with definite unilateral MD were included. All patients received Caloric Test (CT), Video Head Impulse Test (vHIT) and Vestibular Evoked Myogenic Potential (VEMP) test within 7 days after visiting the hospital. Results of these tests were compared between groups.

**Results:**

Most VM patients (64.0%) experienced spontaneous internal vertigo, while most MD patients (66.7%) experienced spontaneous external vertigo. MD patients had more severe vestibular symptoms and autonomic responses compared to VM patients during attacks (*p* =  0.03, *p* = 0.00, respectively). The nystagmus intensity of CT-induced was greater in VM patients than in MD patients (*p* = 0.003). More VM patients had CT intolerance and Central Positional Nystagmus (CPN) compared to MD patients (*p* = 0.002, p = 0.006, respectively). More MD patients had CT(+) and vHIT saccades wave compared to VM patients (*p* < 0.001, *p* = 0.002, respectively). The non-elicitation rate of cervical VEMP was higher, and the ocular VEMP amplitudes were lower in MD patients than in VM patients (*p* = 0.002, *p* = 0.018).

**Conclusions:**

Vestibular symptoms during attacks combined with the results of vestibular function tests may be used to differentiate between VM and MD. The diverse nature of vestibular symptoms (especially internal vertigo), history of motion sickness and CT intolerance may provide clues to the diagnosis of VM, whereas spontaneous external vertigo, CT(+) with vHIT(-), and the presence of saccades may provide clues to the diagnosis of MD.

**Level of evidence:**

4.

## Highlights

• Spontaneous internal vertigo was experienced by most VM patients.
• Most VM patients have high SPV of caloric-induced nystagmus and CT intolerance.
• Spontaneous external vertigo was experienced by most MD patients.
• MD patients mostly have severe vestibular symptoms and autonomic responses.
• Most MD patients show abnormal CT and normal vHIT, with the presence of saccades.

## Introduction

Episodic Vestibular Syndrome (EVS) is a disorder characterized by brief episodes of vertigo, dizziness, and unsteadiness, lasting seconds to several hours, occasionally several days. The two most common EVS are Vestibular Migraine (VM) and Meniere's Disease (MD). Clinically, VM is typically characterized by recurrent vestibular symptoms and attacks with migraine features (migraine, photophobia, phonophobia), with a prevalence of about 1%‒2.7%,^1^ whereas MD as an inner ear disease is typically characterized by recurrent vestibular symptoms and audiological symptoms (fluctuating sensorineural hearing loss, aural fullness and tinnitus), with a prevalence of about 0.2%‒0.5%.^2^

The differential diagnosis of VM from MD is challenging, because 28% of MD patients were reported to have VM symptoms, and 23% of VM patients have MD symptoms.^3^ In clinical practice, VM and MD have tremendous impact on patients' daily life and work activities, and they are central and peripheral vestibular disorders, respectively, reatment methods and prognosis differ between patients with VM and MD, so it is necessary to further differentiate between VM and MD.

However, there is a lack of objective tests that can differentiate the vestibular symptoms between VM and MD patients. A previous study^4^ compared the results of Video Head Impulse Test (vHIT) and Caloric Testing (CT) in patients VM and MD, and found that CT was helpful in differentiating between VM and MD, but the study only evaluated semicircular canal function by vHIT and CT, and did not evaluate the intensity of CT-induced nystagmus and CT intolerance. The results of another study^5^ anayzed the reaults of Vestibular Evoked Myogenic Potential (VEMP) in patients with VM and MD and showed that dysfunction of the saccule was more common in pateints with MD than those with VM. However, only few vestibular function tests (with limited test indexes) were used in the differential diagnosis of VM and MD acorss previous studies.

Therefore, in this study, we explored the clinical characteristics of VM and MD in a multidimensional manner, and compared the results of CT, vHIT and VEMP between patients with VM and MD, so as to find a test battery to identify the two diseases.

## Methods

### Patients

This was an observational, retrospective record review of 102 patients presenting with dizziness who visited the Vertigo Clinic of our hospital from January 2019 to July 2021. Definite VM were diagnosed based on the Barany Society Diagnostic Criteria for VM^6^ (e.g., episodes of vestibular symptoms of moderate or severe intensity and a history of migraine), definite unilateral MD were diagnosed based on the Barany Society Diagnostic Criteria for MD^7^ (e.g., episodic vertigo syndrome, sensorineural hearing loss and fluctuating aural symptoms). All patients received CT, vHIT and VEMP tests within 7 days after visiting the hospital. Routine otologic testing was performed to exclude patients with otologic disease, intracranial space occupying lesions, central nervous system disease, cervical spondylosis, and oculomotor dysfunction.

The study was approved by Peking University Aerospace School of Clinical Medicine (Aerospace Center Hospital). Written informed consent was obtained from all patients.

### Clinical data collection

Data were collected through review of medical records. Clinical data including sex, age, symptoms during attacks, duration of symptoms and accompanying symptoms, neurological examination, and other auxiliary examinations, such as head MRI were collected.

Vestibular symptoms (e.g., predominant symptoms experienced by patients during acute attacks) including internal vertigo (a false sensation of motion of self), external vertigo (a false sensation of spinning and motion of surroundings) and duration of vestibular symptoms, migraine features (migraine, photophobia, phonophobia, and visual aura), auditory symptoms (hearing loss, ear fullness, tinnitus) and autonomic responses were collected.

The severity of vestibular symptoms was assessed using a self-report rating scale of dizziness/vertigo ranging from grades 1 to 5: Grade 1—I feel dizzy, but it has no impact on my daily activities; Grade 2—When I am feeling dizzy, I have to stop what I am doing for a while, the dizziness disappeared quickly, I can resume my activities immediately, I do not have to change any plans due to dizziness; Grade 3—When I am feeling dizzy, I have to stop what I am doing for a while, the dizziness disappeared quickly, I can resume my activities immediately, I have to change some plans due to dizziness; Grade 4—I must exert a great deal of effort to deal with daily activities, but I am often unable to do these things because of dizziness; Grade 5—I am unable to deal with daily activities, and even essential activities are limited.

### Vestibular function tests

#### CT

Nystagmus, eye movements, and the Slow Phase Velocity (SPV) values of CT-induced were recorded with Video-Nystagmography (VNG, Interacoustics, Middelfart, Denmark). Patient in the supine position with 30° head flexion and air irrigator airflow at 50° and 24° within 60 s. Jongkees formula was used to determine Canal Paresis (CP), CP > 25% was defined as unilateral horizontal semicircular canal dysfunction, namely, CT(+). CT intolerance refers to the main symptoms, including obvious nausea, vomiting, palpitations, numbness in hands and feet, and body stiffness.

#### vHIT

vHIT (Interacoustics, Middelfart, Denmark) was used to evaluate the function of the three pairs of semicircular canals. 20 head impulses were delivered in each canal plane in a brief, rapid, passive manner, the angular velocity was 150–250°/s for the horizontal canal impulses, 100–200°/s for the vertical canal impulses, and the amplitude was 10–20°. The vHIT software was used to record the average slow phase Vestibulo-Ocular Reflex (VOR) gain values (the ratio of eye velocity to head velocity at 60 s). The normal VOR gain for the horizontal semicircular canals was between 0.79‒1.20.^8^, ^9^ The presence or absence of overt or covert saccades, and the VOR gain were recorded. A reduction in VOR gain value with the presence of saccades indicated vHIT(+).

#### VEMP

Eclipse evoked potential system (Interacoustics, Middelfart, Denmark) was used to record the response and waveforms for the right and left ears. Normal bilateral amplitude asymmetry ratios were defined as cVEMP ≤ 0.35 and oVEMP ≤ 0.33. The normal VEMP latency values defined by our laboratory were: N18 and P25 cervical VEMP (cVEMP) latencies, N13 and P18 ocular VEMP (oVEMP) latencies. Non-elicitation of waveforms, amplitude asymmetry ratio falling outside of the normal range, and latency prolongation were considered as abnormal VEMP results.

### Statistical analysis

Continuous variables were expressed as mean ± Standard Deviation (SD) or median with Interquartile Range (IQR). The normality of distribution of continuous variables was tested by Kolmogorov-Smirnov test. Comparisons for normally distributed data between groups were performed by using two independent sample *t*-test. The categorical variables were expressed as percentages, and comparisons between groups were performed using the Chi-Square (χ2) test with Yates' continuity correction or Fisher's exact test, as appropriate. All reported p-values are two-tailed, and a *p* < 0.05 was considered statistically significant. All statistical analyses were performed with SPSS 20.0 software.

## Results

### Clinical characteristics of patients with VM and MD

#### Baseline characteristics

In the VM group, there were 14 males and 57 females, with an average age of 44.00 ± 14.44 years (range 12–66 years old). In the MD group, there were 13 males and 18 females, with an average age of 58.50 ± 12.88 years (range 24–76 years old). Migraine features, motion sickness, and Central Positional Nystagmus (CPN) were more common in VM group than MD group (*p* = 0.001, 0.010, 0.002, respectively). Auditory symptoms were more common in MD group than VM group (*p* < 0.001, Table 1).

**Table 1:** Clinical baseline characteristics of VM patients and MD.

	VM (*n* = 71)	MD (*n* = 31)	*p*-value
Sex ratio (male:female)	14 (19.7%):57 (80.3%)	13 (41.9%):18 (58.1%)	*p* = 0.019^a^
migraine features (yes)	71 (100%)	9 (29.0%)	*p* = 0.00^a^
Auditory symptoms (yes)	26 (36.6%)	31 (100%)	*p* = 0.000^a^
History of motion sickness	38 (53.5%)	8 (25.8%)	*p* = 0.010^a^
Central positional nystagmus	28 (39.4%)	2 (6.5%)	*p* = 0.002^a^

a *p* < 0.05.

#### Vestibular symptoms

**Figure 1:**
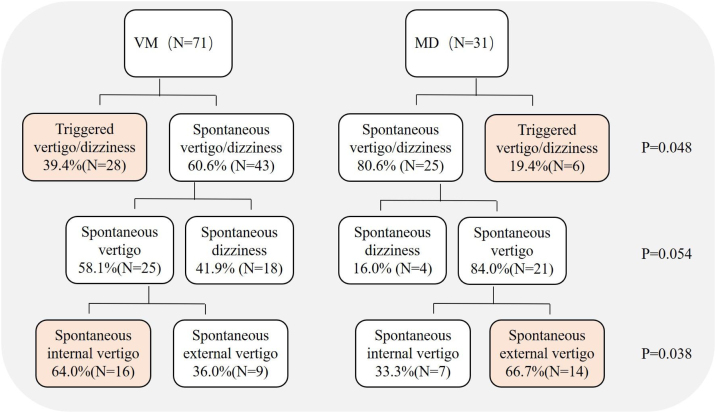
Classification of vestibular symptoms in VM patients and MD.

Spontaneous and triggered dizziness/vertigo were experienced by 60.6% (43/71) and 39.4% (28/71) of VM patients, respectively, which were experienced by 80.6% (25/31) and 19.4% (6/31) of MD patients, respectively, the differences were statistically significant between two groups (Chi-Square test, *p* = 0.048). Further analysis of patients with spontaneous dizziness/vertigo showed that 58.1% (25/43) and 41.9% (18/43) of VM patients experienced spontaneous vertigo and spontaneous dizziness, respectively, 84.0% (21/25) and 16.0% (4/25) of MD patients experienced spontaneous vertigo and spontaneous dizziness, respectively, the differences were not statistically significant between two groups (Chi-Square test, *p* = 0.054). Further classification of spontaneous vertigo showed that 64.0% (16/25) and 36.0% (9/25) of VM patients experienced spontaneous internal and external vertigo, respectively, 33.3% (7/21) and 66.7% (14/21) of MD patients experienced spontaneous internal and external vertigo, respectively, the differences were statistically significant between the two groups (Chi-Square test, *p* = 0.035, Fig. 1).

#### Severity of vestibular symptoms and autonomic responses

MD patients had more severe vestibular symptoms compared to VM patients during attacks (Nonparametric test, *p* = 0.035, Table 2). MD patients also had more severe autonomic responses during attacks (Nonparametric test, *p* < 0.001, Table 3).

**Table 2:** Severity of vestibular symptoms in VM patients and MD during attacks.

Groups	Severity of vestibular symptoms					Z value	*p*-value
	1 Grade	2 Grade	3 Grade	4 Grade	5 Grade		
VM (*n* = 71)	2	32	33	3	1	−2.958	0.03
MD (*n* = 31)	0	7	17	4	3

**Table 3:** Severity of autonomic responses in VM patients and MD during attacks.

Groups	Severity of autonomic response			Z value	*p*-value
	None	Mild	Severe		
VM (*n* = 71)	27	19	15	−4.082	<0.001
MD (*n* = 31)	5	3	23		

The severity of autonomic response: none, absence of nausea and vomiting; mild, nausea; severe, vomiting.

#### Duration of vestibular symptoms

**Figure 2:**
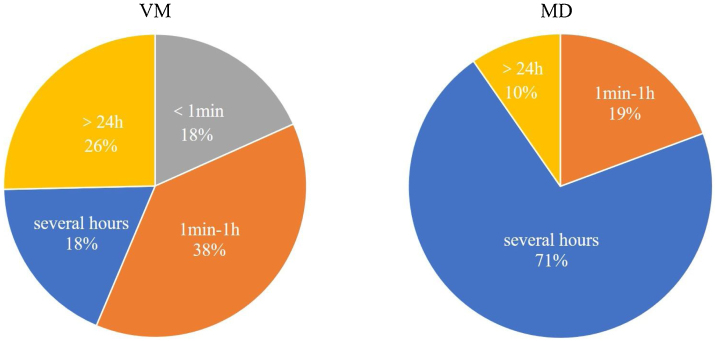
The duration of vestibular symptoms in VM patients and MD. The duration of vestibular symptoms was divided into: <1 min, 1 min–1 h, hours, and >24 h.

The duration of vestibular symptoms in VM patients varied widely, the symptoms lasted less than 1 min in 18.3% (13/71) of patients, between 1 min and 1 h in 38.0% (27/71) of patients, several hours in 18.3% (13/71) of patients, more than 24 h in 25.4% (18/71) of patients. The duration of vestibular symptoms in MD patients usually lasted several hours, between 1 min and 1 h in 19.4% (6/31), several hours in 71.0% (22/31), and more than 24 h in 9.7% (3/31). The differences were statistically significant between two groups (*p* < 0.001, Fig. 2).

#### Vestibular function test results

##### CT

**Figure 3:**
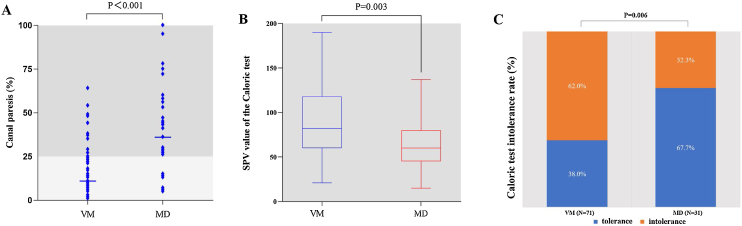
Comparison of CT results between VM patients and MD. (A) Horizontal semicircular canal function in VM and MD patients. (B) The SPV of caloric-induced nystagmus in VM and MD patients. (C) The incidence of CT intolerance in VM and MD patients.

The incidence of CT(+) was higher in MD patients than in VM patients (77.4% [24/31] vs. 21.1% [15/71], Chi-Square test, *p* < 0.001, Fig. 3A). The nystagmus intensity of CT-induced (the SPV values of CT-induced nystagmus) was greater in VM patients than in MD patients (89.14 ± 39.88 vs. 65.23 ± 27.82, *p* = 0.003, Fig. 3B). The incidence of CT intolerance was significantly higher in VM patients than in MD patients (62.0% [44/71] vs. 32.3% [10/31], Chi-Square test, *p* = 0.006, Fig. 3C).

##### vHIT

**Figure 4:**
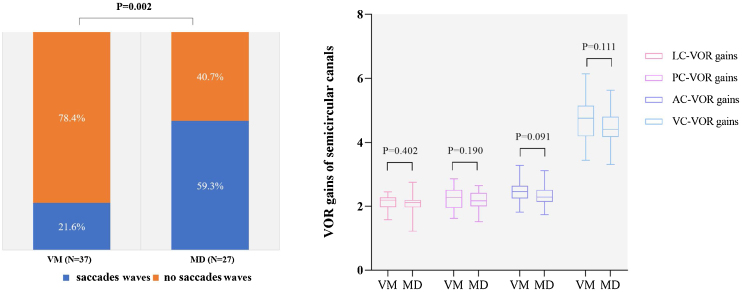
Comparison of vHIT results between VM patients and MD. (A) The presence or absence of saccades. (B) VOR gains of all semicircular canals. LC, Horizontal Canal; PC, Posterior Canal; AC, Anterior Canal; VC, Vertical Canal.

Fewer VM patients had overt and covert saccades in the horizontal semicircular canal compared with MD patients (21.6% [8/37] vs. 59.3% [16/27], Chi-Square test, *p* = 0.002, Fig. 4A). No statistically significant difference was found in VOR gains of all semicircular canals between the two groups (*p* > 0.05, Fig. 4B). 2.7% (1/37) of VM and 3.7% (1/27) of MD patients had abnormal vHIT (Chi-square test, *p* > 0.05).

##### The combined results of CT and vHIT

CT(−) with vHIT(−) was the most common pattern in VM group, which was observed more frequently when compared with MD group (78.4% [29/37] vs. 18.5% [5/27], Chi-Square test, *p* < 0.001, Table 4), whereas CT(+) with vHITT(−) was the most common pattern in the MD group (18.9% [7/37) vs. 77.8% [21/27], Chi-Square test, *p* < 0.001, Table 4).

**Table 4:** Comparison of the combined results of CT and vHIT between VM patients and MD.

Group	CT(−) vHIT(−)	CT(−) vHIT(+)	CT(+) vHIT(−)	CT(+) vHIT(+)
VM (*n* = 37)	29 (78.4%)	0 (0.00%)	7 (18.9%)	1 (2.7%)
MD (*n* = 27)	5 (18.5%)	0 (0.00%)	21 (77.8%)	1 (3.7%)
*p*-value	<0.001		<0.001	1.000

##### VMEP

**Figure 5:**
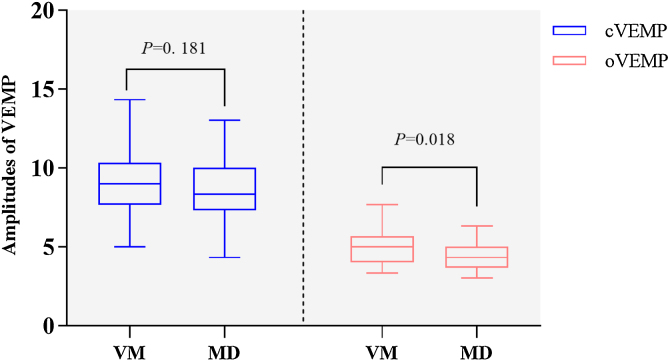
Comparison of VEMP results between VM patients and MD.

There were no significant differences in the abnormal rates of oVEMP and cVEMP between the two groups (oVEMP, 19.2% [5/26] vs. 32.1% [9/28], Chi-Square test, *p* = 0.279; cVEMP, 8.5% [5/27] vs. 30.8% [8/26], Chi-Square test, *p* = 0.300). However, the non-elicitation rate of cVEMP was higher in the MD group than in the VM group (19.2% [5/26] vs. 0.0% [0/27], Fisher's Exact test, *p* = 0.002). There were significant differences in oVEMP amplitude between VM and MD groups (5.00 [4.00–5.67] vs. 4.33 [3.67–5.00], Nonparametric test, *p* = 0.018, Fig. 5), no statistically significant difference in cVEMP amplitude between the two groups (9.00 ± 1.96 vs. 8.33 ± 1.85, *t*-test, *p* = 0.181, Fig. 5).

## Discussion

### Vestibular symptoms in VM and MD

Few studies have been conducted to differentiate the vestibular symptoms between VM and MD patients. In this study, we found that the nature of vestibular symptoms during attacks in VM patients was diverse, with both spontaneous and triggered dizziness/vertigo being common. Numerous studies have found that spontaneous vertigo accounted for 20%‒85% of VM patients, head motion intolerance and positional vertigo accounted for 20%‒85% and 18%‒60% of VM pateints, respectively.^10^, ^11^ In contrast, spontaneous vertigo is the most common complaint among MD patients. In terms of internal/external vertigo, we found that VM patients experienced spontaneous internal vertigo, whereas MD patients experienced spontaneous external vertigo. Our results are consistent with the finding from a prospective multi-center study.^12^ showing that internal vertigo was more common than external vertigo in VM patients. We also found that the duration of vestibular symptoms in VM patients varied widely, while MD patients lasted mainly for several hours. Additionally, the results of this study showed that MD patients had more severe vestibular symptoms and autonomic responses than those with VM during attacks. A previous study^12^ showed VM patients often experience nausea (59.9%), and rarely experience vomiting (17.8%). MD patients complain of more severe vestibular symptoms than VM patients during the attacks, our results further confirm that VM is a central vestibular disorder, and MD is a peripheral vestibular disorder, and indicate that the diverse nature of vestibular symptom episodes (especially internal vertigo) may provide clues to the diagnosis of VM. In contrast, external vertigo, vestibular symptoms lasing several hours, and more severe vestibular symptoms and autonomic responses may provide clues to the diagnosis of MD.

### Accompanying symptoms in VM and MD

In this study, we found that 36.6% of VM patients had auditory symptoms, and 29.0% of MD patients had migraine features. The overlap of symptoms during vertigo attacks in MD and VM makes it more difficult to distinguish between them. We also found that motion sickness was more common in VM patients than in MD patients. The result is consistent with previous studies showing that VM patients were more vulnerable to motion sickness,^10^, ^13^, ^14^ indicating that VM patients exhibit vestibular hypersensitivity and visual-vestibular mismatch. Therefore, motion sickness may provide clues to the diagnosis of VM.

### Vestibular function in VM and MD

Results of this study showed that the nystagmus intensity of CT-induced was greater in VM patients than that in MD patients. Similar results were obtained by a previous study.^15^ However, another study^13^ showed that the nystagmus intensity of CT-induced was not different between VM and MD patients. The results of the present study also showed that compared with MD patients, VM patients were more likely to develop CT intolerance, and intolerance-related symptoms may persist for several hours after completion of CT. Previous studies^13^, ^16^, ^17^ also found that CT intolerance occurred more frequently in VM patients, and the mechanism may be related to the increase in VOR time constant caused by hypersensitivity of vestibular system.^18^, ^19^, ^20^ VM patients were more likely to develop CT-induced nystagmus and CT intolerance, we hypothesized that vestibular hypersensitivity may be a possible mechanism for VM, and vestibular hypersensitivity is possibly caused by the disinhibition of sensorimotor network and vestibular cortical network.^17^

In this study, we found that the majority of MD patients had CT(+) and vHIT(−), the result is consistent with previous studies.^21^, ^22^ The mechanism for dissociation between CT and vHIT results is believed to be endolymphatic hydrops.^21^, ^23^ Endolymphatic hydrops expands the volume of endolymphatic duct, causing convective currents between low- and high-density endolymph, resulting in pressure gradient created near the crista ampullaris and the reduction of the buoyant force, which counteract the hydrostatic pressure acting on the crista ampullaris, thus weakening the degree of hair cell deflection on the crista ampullaris. Therefore, MD patients had CT(+). However, since the volume or shape of the entire bony labyrinth does not change, there is no change in the stimulation velocity produced by vHIT, the vHIT gain was normal in MD patients. A previous study^24^ found that in MD patients with normal vHIT gain, the reduction in caloric response was correlated with the degree of endolymphatic hydrops. In this study, we also found that the majority of MD patients had normal vHIT gain but exhibited horizontal canal saccades, suggesting that in addition to the impairment of low-frequency semicircular canal function, MD patients may also have mild impairment of high-frequency semicircular canal function. Several studies have shown that during vHIT, saccades were usually found in MD patients.^25^, ^26^ A study^27^ also showed that the 1st saccade velocity on the affected side could indicate the duration and severity of MD.

In this study, the majority of VM patients showed CT(−) and vHIT(−), 21.1% of VM patients showed CT(+), and 2.7% showed vHIT(+). In previous studies, the abnormal rates of CT and vHIT in VM patients were reported to be 8%‒22 %,^28^ and 9%‒11%,^28^, ^29^ respectively. This evidence seemed to support that peripheral vestibular deficit also contributes to the pathogenesis of VM.

Therefore, a battery of CT and vHIT can be used to differentiate between MD and VM. Multiple vestibular function test indexes can be evaluated. For example, during CT, CP values, the intensity of CT-induced nystagmus, and CT intolerance can be assessed. The stronger intensity of CT-induced nystagmus and CT intolerance may provide clues to the diagnosis of VM, while CT(+) with vHIT(−), and the presence of overt and covert saccades may provide clues to the diagnosis of MD.

VEMP has been used to assist in differentiating between VM and MD, but the results are controversial.^5^, ^30^ A prospective cohort study showed that VEMP were not able to discriminate between VM and MD, but MD patients showed reduced oVEMP amplitude.^31^ In this study, oVEMP amplitude was lower in MD patients than in VM patients, but the non-elicitation rate of cVEMP was higher in MD patients than that in VM patients. We hypothesized that most MD patients have saccular dysfunction.

Additionally, 29.6% (21/71) of VM patients showed CPN. We hypothesized that the vestibular nuclei is activated by brainstem regions associated with migraine (e.g., locus coeruleus; dorsal raphe nucleus) during VM attacks, inhibition of the cerebellar nodulus and uvula is no longer sufficient to maintain stability, so changes in the head position often result in positional nystagmus and vertigo.^19^, ^32^ Brain dysfunction may be a mechanism for VM.

### Limitation

First, this is a sigle-center, retrospective study with a small sample size, future studies with larger sample sizes are needed to confirm our findings. Second, the present did not performe audiologic tests in pateints with VM and MD, it is worth further investigating whether a battery of audiologic tests can be used to distinguish these between the two disorders.

## Conclusions

Vestibular symptoms during attacks combined with the vestibular function tests may be used to differentiate between VM and MD. The diverse nature of vestibular symptoms (especially internal vertigo), history of motion sickness and CT intolerance may provide clues to the diagnosis of VM. Vestibular hypersensitivity and brain dysfunction are speculated to be the possible mechanisms of VM. Spontaneous external vertigo, CT(+) with vHIT(−), and the present of saccades may provide clues to the diagnosis of MD.

## Authors’ contributions

Xu Yang contributed to the conception and design of the study. Ning Song, Xiang Li, Tongtong Zhao, Menglu Zhang collected the clinical data. Yuexia Wu, Xia Ling analyzed the results and drafted and corrected the manuscript. All authors contributed to the article and approved the submitted version.

## Funding

This study was supported by Aerospace Center Hospital (HP2021-03-50703).

## Conflicts of interest

The authors declare no conflicts of interest.
